# Epigenetic Modulation of Chromatin States and Gene Expression by G-Quadruplex Structures

**DOI:** 10.3390/ijms21114172

**Published:** 2020-06-11

**Authors:** Chiara Reina, Vincenzo Cavalieri

**Affiliations:** 1 Department of Health Promotion, Mother and Child Care, Internal Medicine and Medical Specialties (PROMISE), University of Palermo, 90127 Palermo, Italy; chiara.reina01@unipa.it; 2 Department of Biological, Chemical and Pharmaceutical Sciences and Technologies (STEBICEF), University of Palermo, 90128 Palermo, Italy

**Keywords:** G-quadruplex, G-quartet, epigenetics, DNA bases modifications, histone post-translational modifications, histone-modifying activities, nucleosome remodeling, chromatin architecture, post-transcriptional regulation, noncoding RNA

## Abstract

G-quadruplexes are four-stranded helical nucleic acid structures formed by guanine-rich sequences. A considerable number of studies have revealed that these noncanonical structural motifs are widespread throughout the genome and transcriptome of numerous organisms, including humans. In particular, G-quadruplexes occupy strategic locations in genomic DNA and both coding and noncoding RNA molecules, being involved in many essential cellular and organismal functions. In this review, we first outline the fundamental structural features of G-quadruplexes and then focus on the concept that these DNA and RNA structures convey a distinctive layer of epigenetic information that is critical for the complex regulation, either positive or negative, of biological activities in different contexts. In this framework, we summarize and discuss the proposed mechanisms underlying the functions of G-quadruplexes and their interacting factors. Furthermore, we give special emphasis to the interplay between G-quadruplex formation/disruption and other epigenetic marks, including biochemical modifications of DNA bases and histones, nucleosome positioning, and three-dimensional organization of chromatin. Finally, epigenetic roles of RNA G-quadruplexes in post-transcriptional regulation of gene expression are also discussed. Undoubtedly, the issues addressed in this review take on particular importance in the field of comparative epigenetics, as well as in translational research.

## 1. Introduction

More than one century ago, seminal observations from Phoebus Levene’s laboratory revealed that guanosine forms polycrystalline gels at high concentrations, providing the first indirect evidence that guanine-rich sequences in nucleic acids may form higher-order structures [1]. Curiously, many authors have attributed the achievement of this discovery to the Scandinavian biochemist Ivar Bang, who described a similar gelling process one year after Levene’s paper, using aqueous solutions of guanylic acid isolated from ox pancreas [2].

More than five decades later, crystallographic analysis of guanosine-5′-monophosphate gels has provided the structural explanation of the jellification phenomenon, revealing the association of guanine bases in tetrameric arrays (referred to as G-tetrads) via non-Watson–Crick base pairing [3]. Further X-ray fiber diffraction and biophysical studies on guanine-rich polynucleotides have confirmed and extended these findings, paving the way for the concept of G-quadruplex, a four-stranded continuous helix whose geometric formalism is fully consistent with the G-tetrad structural motif [4,5,6].

Since then, a very large collection of evidence has identified these structures in nucleic acid sequences of a wide spectrum of organisms, highlighting their involvement in the regulation of various key cellular and organismal processes, including embryogenesis, genome maintenance, replication and expression [7,8,9,10,11,12,13,14,15,16]. In this review, we will focus on the epigenetic roles played by both DNA and RNA G-quadruplexes in the modulation of chromatin states and gene expression. 

## 2. Structural Features of G-Quadruplexes and Their Occurrence in Biological Contexts

**Figure 1:**
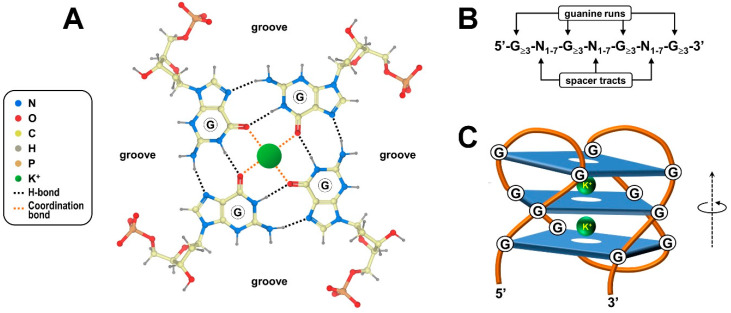
Chemical structures of G-quartet and G-quadruplex. (**A**) Structural arrangement of the G-quartet, highlighting the hydrogen bonding network between the Hoogsteen and Watson–Crick faces of the coplanar guanine bases. The attached deoxyribose sugars are shown together with a centrally placed metal ion. (**B**) The conventional consensus sequence for a G-quadruplex. (**C**) Side view of the schematic diagram showing an intramolecular antiparallel G-quadruplex formed by the stacking of three G-quartets. Strand polarity and anticlockwise rotation are indicated.

G-quadruplexes, henceforth referred to as G4s, are four-stranded knot-like structures dynamically folded in guanine-rich regions of nucleic acids. As mentioned, the building block of these noncanonical structures is the G-tetrad or G-quartet, a coplanar cyclic array of four adjacent guanylic nucleotides held together by a network of hydrogen bonds at the edges of the resulting square platform (Figure 1A). In particular, the guanine purine rings simultaneously accomplish hydrogen bond donor and acceptor functionalities, yielding eight Hoogsteen-type interactions per G-quartet [3,17]. In this tetrameric arrangement, distinct combinations of *anti* and *syn* conformations, defined by torsion angles around the N-glycosidic bond that connects the guanine base to the sugar, directly determine the parallel/antiparallel directionality of the phosphodiester backbone of each of the four participating polynucleotide strands [18,19].

Since the prototypical G4-forming consensus sequence 5′-G_≥3_-N_1–7_-G_≥3_-N_1–7_-G_≥3_-N_1–7_-G_≥3_-3′ includes four runs of at least three consecutive guanines (Figure 1B), a given G4 generally, but not always, contains a minimum of three G-quartet layers stacked upon one another by virtue of electron interactions between π orbitals from their aromatic surfaces (Figure 1C) [20,21]. Lone pair electrons at the oxygen atom of each guanine carbonyl group lie in the central core of adjoining G-quartets, creating an electronegative axial tunnel running through the G4 stack (Figure 1C) [22,23]. In this context, the symmetric bipyramidal antiprismatic arrangement for eight oxygen atoms juxtaposed at each level of the stack allows coordination of monovalent alkali metal cations that, in turn, impart further stability to the G4 structure (Figure 1C) [24]. In the cellular environment, potassium ions are preferentially coordinated because they exist in the highest concentration and give the best size match for the accommodation inside the G4 central channel [24,25].

Worth mentioning, although G4s are commonly depicted as having a straight three-dimensional morphology, they are instead helical structures displaying rotational symmetry and four grooves varying in width in the spaces between the four guanine-rich strands (Figure 1) [26]. In the overall G4 structure, G-quartets are stacked perpendicularly to the helix axis, and continuous stretches of guanines establishing the stacked G-quartets are connected by loops of spacer tracts varying in length and nucleotide composition (Figure 1C) [18]. The structural uniformity of the G4 stack is tolerant of bulging out single unpaired bases or embedding them into the core structure, which broadens the definition of G4-forming sequences, as well as the specific nomenclature of G4 themselves [27]. Moreover, G4s can arise from a single polynucleotide chain of DNA or RNA containing an adequate number of guanine-run stretches or can alternatively embrace distinct guanine-rich regions belonging to multiple (either two, three or four) nucleic acid chains [28,29,30]. Adding further complexity, stacking of intra-/intermolecular G-quartets may produce G-wires, high-order thread-like superstructures exhibiting peculiar periodicity and physical properties that are not found in basic G4s [31,32].

In sum, it can be argued that the repertoire of G4 molecular architectures displays extensive geometry and conformational polymorphism, comprehensively according to the variability of the aforementioned intrinsic structural parameters [33,34,35,36,37]. Additional sources for the high topological divergence are given by extrinsic factors, including chemical modification and pH-driven protonation/de-protonation of bases [38,39,40,41], molecular crowding [42], and the presence of chaperone molecules [43,44].

Genomewide computational screenings for sequence motifs that have the propensity to form G4, as well as G4-specific chromatin immunoprecipitation coupled with high-throughput sequencing analysis, have revealed widespread G4 occurrence in a large number of species belonging to all the kingdoms of life, and in diverse ribo- and deoxyribo-viruses [45,46,47,48,49,50,51,52,53,54,55]. These studies have coherently indicated that the global amount of genomic G4s displays considerable variation across species, roughly according to size and guanine-richness of their genome, but not to evolutionary distances [56,57,58,59,60]. Furthermore, they unveiled that G4s are not randomly scattered across the genome, being rather biased toward telomeric sequences, satellite DNA, noncoding transcription units, and gene *cis*-regulatory regions [61,62,63,64]. Intriguingly, comparative inspections have also highlighted similarities across diverse organisms and species-specific trends of G4 distribution in conserved genomic portions [57,58,59,65]. For example, strong G4 enrichment in gene promoters has been reported in higher vertebrates, including humans, while preferential enrichment in noncoding transcription units has been detected in other metazoans such as the nematode worm *Caenorhabditis elegans* and zebrafish (*Danio rerio*) [57]. Unfortunately, it remains poorly understood whether all of the G4s emerging from these stimulating studies actually form in vivo, especially in light of the fact that only a very modest fraction of them have been experimentally validated by immune-fluorescent visualization in different types of living cells [66,67,68,69]. Beyond this direct line of evidence provided by employing G4-specific antibodies generated by independent groups, G4 formation in vivo has been indirectly justified by alternative approaches, such as “in-cell” NMR spectroscopy [70,71], and by the recent identification and functional characterization of G4-specific DNA and RNA helicases from various organisms [72,73,74,75,76,77,78].

In fact, although G4s are generally conceived as energetically favorable and highly stable structures under physiological conditions, their assembly in vivo often requires preliminary local chromatin dismantling and DNA double-helix denaturation. This occurs especially during biological processes, such as replication and transcription, that transiently expose single-strand DNA segments. Once formed, G4s on both DNA and RNA molecules can contribute to the regulation of fundamental cell functions, as described in the following sections.

## 3. Involvement of G4 Structures in Epigenetic Processes

Based on our actual understanding of epigenetics, an epigenetic trait is broadly intended as a reversible molecular modification associated with changes in gene expression in the absence of variation in genomic DNA sequence, occurring in the course of adaptive responses of a given cell/organism to environmental influence [79,80]. There is growing experimental evidence indicating that G4s entirely fulfill this definition, being, in principle, interconverting DNA structural conformations dynamically adopted by peculiar genome segments. The occurrence of G4 structures in regulatory regions such as promoters and enhancers may influence gene expression either positively or negatively, thereby provoking transcriptome changes [81,82]. Folding of G4 structures is differentially affected, either directly or indirectly, by disparate environmental cues, including dietary molecules and epigenetic drugs [83,84,85,86,87,88]. In addition, G4 structures and other established epigenetic modifications frequently go side by side, and they harmoniously affect each other in many fascinating ways, reshaping genome transcriptional outputs.

### 3.1. Interplay between G4 Structures and Epigenetic Modifications of DNA Bases

**Figure 2:**
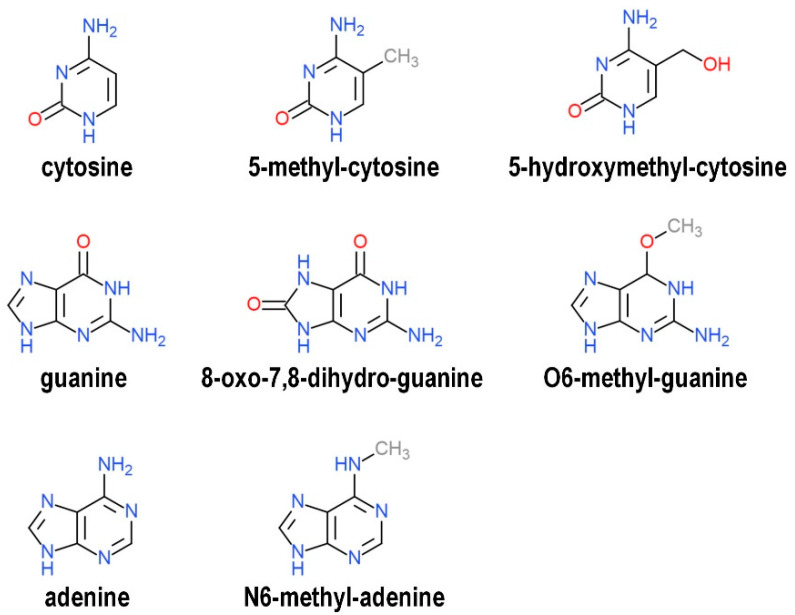
Chemical structure of canonical and biochemically modified nitrogenous bases discussed in this review.

Stability of a conventional G4 structure may be affected by epigenetic biochemical modifications of sequences either forming or flanking the G4 motif in the same DNA strand, such as methylation at carbon 5 on the cytosine pyrimidine ring (5meC, Figure 2). In fact, G4s exhibit much higher thermal stability when sandwiched by stacking forces provided by nearby 5meC-5meC+ base pairs formed by N3-protonation of cytosine under physiological-like conditions [89,90,91]. In certain exceptional cases, however, methylated G4s appear to be less thermally stable than their unmethylated equivalents, suggesting that the influence of methylation on G4 thermodynamic properties is strictly dependent on the DNA sequence being investigated [92]. It can be argued that the connection between G4 stability and cytosine methylation could have relevant consequences in vivo, especially because DNA methylation generally results in a reduction of chromatin accessibility and transcriptional activity. In this respect, recent genomewide approaches have shown that the occurrence of intrinsically stable G4s and 5meC is inversely related at CpG island promoters of different human cell lines and tissues, as well as in peripheral blood samples from distinct individuals, strongly suggesting that G4s are epigenetic marks of active hypomethylated chromatin [93,94,95]. This finding is further supported and extended by separate mechanistic evidence indicating that (i) G4 structures, once formed, avidly interact with DNA methyltransferase (DNMT) enzymes both in vitro and in vivo, and that (ii) G4-dependent recruitment of DNMT1 at lowly methylated CpG islands locally results in significant inhibition of DNMT1 enzymatic activity [95,96]. Taken together, these findings point to the coordinated contribution of two distinct classes of epigenetic players, G4 structures and the enzymes that establish and maintain deoxycytidine methylation, in shaping the methylome.

It is commonly accepted that G4 structures formed on sequences juxtaposed to the gene core promoters may inhibit transcriptional activity by acting as a steric block to RNA polymerase recruitment, an effect that is exacerbated when the G4-forming regions undergo cytosine methylation [97]. Along with this negative impact on transcription, the co-occurrence of G4 formation and cytosine methylation can instead give a stimulatory contribution to gene expression. To cite an example, cytosine hypomethylation in the core promoter region, coupled with methylation in a specific CpG in the first exon, favors *hTERT* gene expression in tumor cells [98,99]. In fact, exonic 5meC triggers the formation of a stable G4 structure that, in turn, prevents the binding of the CTCF factor, otherwise involved in *hTERT* transcription repression [99,100].

A major route to CpG demethylation in metazoans involves the sequential enzymatic conversion of 5meC into the progressively higher oxidation states of 5-hydroxymethyl-, 5-formyl-, and 5-carboxyl-cytosine until the base is excised and replaced by an unmodified cytosine [101,102]. Intriguingly, emerging evidence suggests that the oxidation products of 5meC could reflect distinctive epigenetic modifications associated with differential genomic density and specific biological functions. For example, transcriptional activity is negatively correlated with 5meC but positively correlated with 5-hydroxymethyl-cytosine (5hmeC, Figure 2) [103,104]. This is probably due to the poor binding affinity of DNMT1 toward 5hmC, suggesting that 5hmC, as opposite to 5meC, could promote DNA demethylation by excluding DNMT1 from CpG islands [105]. In human stem cells, a very small fraction of G4s harbors 5hmeC in loop regions, and the presence of this modification does not markedly affect G4 formation or stability [106,107]. On the other hand, it has been shown that the oxidized derivatives of cytosine dynamically recruit distinct sets of regulatory proteins in differentiating mouse embryonic stem cells, suggesting that G4-associated cytosine modifications epigenetically influence the propensity of G4 structures to be recognized by DNA-binding effector proteins [108]. Recent studies focused on the G4 from the *vegf* promoter have revealed that this is indeed the case, where the presence of 5meC significantly decreases the binding ability of the VEGF165 protein, while 5hmeC specifically abrogates nucleolin recruitment [107,109,110].

Epigenetic variations reverberating on G4 structure stability can also be inflicted by environmental stress endured by individual cells or organisms. A pertaining example is provided by the establishment of guanine oxidation, which is induced by reactive oxygen species from both exogenous and endogenous origins [111]. At the DNA level, the redox potential of the guanine heterocycle is particularly low, and it further decreases proportionally when a rising number of adjacent guanine bases stack upon one another [112,113]. It follows that guanine runs embedded in G4-forming sequences are the most prone to oxidation within eukaryotic genomes, where 8-oxo-7,8-dihydro-guanine (8oxoG, Figure 2) is the oxidation product found in the highest yield. To give an idea of scale, 8oxoG is present at a very low frequency in murine embryonic stem cells and adult cortex tissue, being more than three orders of magnitude smaller in concentration than 5meC in the genome [114].

From a structural perspective, the presence of 8oxoG impacts either positively or negatively on G4 integrity, depending on the exact position occupied within the quadruplex [115,116]. For instance, the substitution of a guanine with 8oxoG at the middle tetrad of a conventional G4 disrupts both the Hoogsteen and stacking interactions, thereby hindering G4 folding. Nonetheless, divergent G4-forming sequences, containing excess guanines in their G runs, and/or more than four G runs, more broadly tolerate oxidative modifications, being able to rearrange into alternative topologies in which 8oxoG is looped out and the overall structural integrity is maintained [117,118]. In the case of G4s occurring in promoter sequences, such as an 8oxoG-driven topology switch, this leads to an upregulation of gene expression during oxidative stress. Based on published evidence, this purely epigenetic mechanism is most probably achieved by the recruitment of OGG1, a DNA glycosylase involved in the reversal of guanine oxidation [119,120]. Following specific recognition of 8oxoG, OGG1 would indeed facilitate the assembly of an effector complex that could contain transcription activators, heterogeneous nuclear ribonucleoprotein particles, and RNA polymerase II for the induction of gene transcription [121,122,123,124,125,126]. According to recent models, the abasic site yielded by OGG1-dependent release of 8oxoG would concomitantly favor the structural switch from duplex DNA to G4 and recruitment of a complex, including the apurinic-apyrimidinic endonuclease 1 (APE1) and other partners such as HIF1-α or the RNA polymerase II. The G4 structural context, however, would severely attenuate the endonuclease activity of APE1, thus allowing transcription activation driven by HIF1-α or direct positioning of RNA polymerase II [126]. A differing model suggests that the base excision repair of 8oxoG embedded into G4 would stimulate gene transcription after returning the sequence back to the wild-type state by means of enhanced recruitment of MYC-associated zinc-finger transcription factor and heterogeneous nuclear ribonucleoprotein A1 [126]. Further studies are needed to address the exact choreography of events regarding the reversal of guanine oxidation located in G4-forming sequences on gene promoters and the upregulation of gene expression.

Much less is known about the relationship between the formation or function of G4s and other biochemical modifications of DNA bases. In this regard, the O6-methyl-deoxyguanine (6meG, Figure 2) adduct is formed in response to alkylating environmental pollutants and enzymatically reversed by O6-alkylguanine DNA alkyltransferase [127]. The presence of this atypical modification significantly weakens the overall G4 structure because 6meG is flipped out from the stacked G-quartets and, therefore, prevented from participating in metal cation coordination [128]. Similarly, it has been recently shown that the presence of N6-methyl-deoxyadenosine (6meA, Figure 2) in an unconventional G4-forming sequence from the human *c-kit* gene is detrimental to G4 folding in vitro, probably due to disruption of Watson–Crick base pairing resulting from the preference for the *syn* conformation adopted by 6meA [129,130,131,132]. Functional findings in vivo need to be addressed since 6meA is widely distributed, although with low abundance, across the eukaryotic genome and predominantly within genomic deserts [133].

### 3.2. G4 Structures and the Histone Epigenetic Machinery

**Figure 3:**
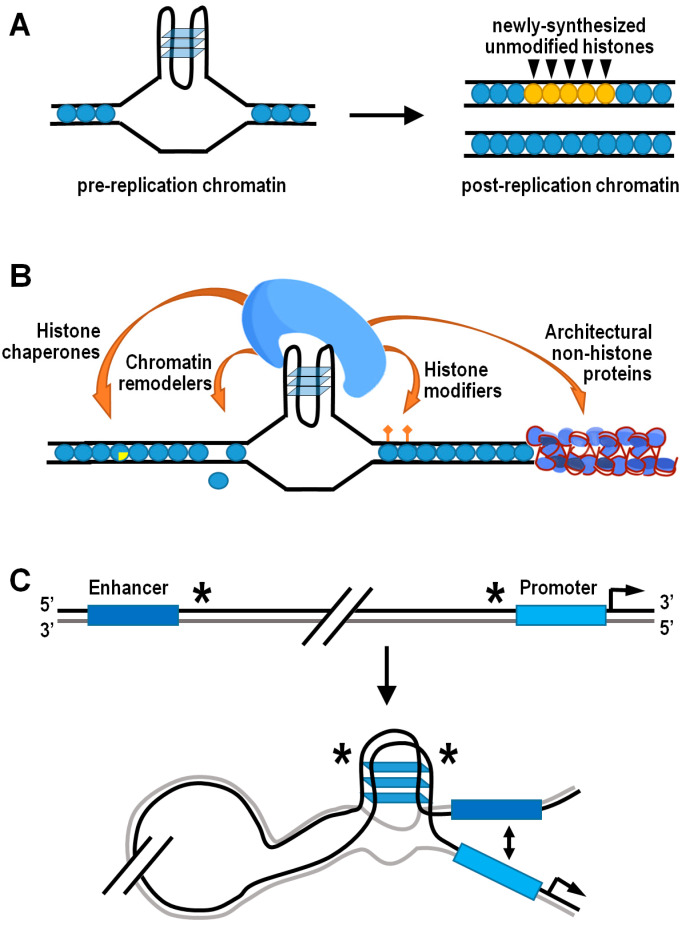
Interplay between G4 structures and the epigenetic machinery. (**A**) Schematic drawing indicating loss of histone epigenetic memory following DNA replication at sites with G4 structures in cells lacking either REV1 or FANCJ. (**B**) Mechanistic model showing that G4 structures can act as docking sites for several classes of epigenetic players. (**C**) Schematic model of enhancer–promoter interaction mediated by an intermolecular G4 structure formed by two distinct half G4s (indicated by asterisks).

A fundamental source of epigenetic information is stored on nucleosomes, in each of four core histones H2A, H2B, H3, and H4 [134,135,136,137,138]. Accurate propagation of the epigenetic information residing into nucleosomes is strictly dependent upon local recycling of modified parental histones to daughter chromatin by histone chaperones during active DNA replication [139,140]. In this respect, structured G4s represent physical obstacles to the replisome progression, and their unfolding is ensured by a number of helicases and/or specialized DNA polymerases involved in translesion synthesis [141,142,143,144]. However, when the unwinding of G4 structures is temporarily delayed, the corresponding genomic regions are bypassed and gaps are completed by postreplicative mechanisms, leading to preferential incorporation of newly synthesized histones that are devoid of parental modifications [145]. Therefore, the G4-dependent decoupling of parental histone recycling from DNA replication ultimately disrupts the local inheritance of epigenetic transmission. This effect has been formerly documented in avian DT40 cells lacking either the Y-family DNA polymerase REV1 or the RecQ-family helicase FANCJ [13,146,147,148]. In both cases, altered transcription of specific loci was well correlated with anomalous histone mark patterns in the vicinity of potential G4-forming sequences (Figure 3A). Strikingly, artificial insertion of a G4-forming sequence into the first intron of the silent lysozyme C (lysC) gene resulted in derepression of lysC in REV1 deficient cells, further highlighting the need for G4-structure processing in the maintenance of histone epigenetic memory and regulation of gene expression [13].

Formation of G4 structures may also provide the docking site for effector protein complexes horboring histone-modifying activities (Figure 3B). For instance, the TLS/FUS/KMT5C ternary complex is able to bind both RNA and DNA G4 structures at telomeric chromatin, where KMT5C specifically trimethylates the lysine 20 residue of nucleosomal histone H4, thus favoring chromatin condensation [149]. Contrarily, the H4K20-specific histone demethylase PHF8 has been associated with G4-containing promoters of genes located in open chromatin and expressed at a significant level [150]. Another well-established example is the REST/coREST repressor complex, which conveys the histone H3K4-specific demethylase LSD1 at peculiar chromatin locations containing G4 structures, including the p21 and hTERT gene promoters [151,152,153,154]. More recently, outcomes from a high-throughput screening assay in vitro added further insights on the interaction between topologically different intramolecular G4 DNA structures and human epigenetic proteins immobilized on microarrays [155]. Top significant hits resulting from this analysis indeed included the peptidylarginine deiminase PADi4, which converts both arginine and monomethyl-arginine histone residues to citrulline [156], and the scaffolding protein ASXL1, which binds to chromatin and recruits polycomb repressive complex 2 (PRC2), thus favoring the locus-specific accumulation of H3K27me3 [157,158]. It is noteworthy that G4 structures in nascent chromatin-associated RNAs are involved in the temporal regulation of PRC2 occupancy at its target genes, as they evict PRC2 from nucleosome particles and inhibit its methyltransferase activity [159,160].

Structured G4s, both in DNA and chromatin-associated RNA, may locally control chromatin compactness and/or composition by directly attracting classic chromatin remodeler and histone chaperones such as SWI/SNF, NuRD, FACT, and BRD3, which are renowned players in nucleosome disassembly/reassembly processes [155]. For example, G4-dependent recruitment of the complex containing the SWI/SNF-like chromatin remodeler ATRX and DAXX histone chaperone permits deposition/exchange of the H3.3 histone variant, which is subsequently targeted for K9 trimethylation to establish a heterochromatic state at telomeric and pericentromeric regions [161,162]. In other chromatin contexts, the FACT-like histone chaperone nucleolin both binds and stabilizes G4 structures, promoting the remodeling of nucleosomes containing macroH2A, and the removal of H2A–H2B dimers from assembled nucleosomes [163,164,165].

As a further possibility, G4s can impact on chromatin dynamics by engaging architectural nonhistone proteins, including some members of the HMG-B and -N families, which alter the pattern of histone modifications and modulate the binding of linker histones to chromatin [166,167,168]. For example, the biological outcome of the HMGB1-mediated G4 stabilization in the promoter of the human kras gene is transcriptional silencing, and the importance of HMGB1 in this mechanism is further supported by the increase in kras gene expression induced following HMGB1 knockdown [169]. A complementary role to HMGB1 could be played by the poly ADP-ribose polymerase 1 (PARP1) protein, which has been positively associated with G4 formation and kras gene expression. In fact, in its activated form, PARP1 specifically binds G4s at the murine kras gene promoter, where it locally behaves as a histone chaperone for chromatin relaxation, triggering nucleosome eviction by histone PARylation [170,171].

On a whole-genome scale, the coalescence of G4 structures and architectural proteins has further relevant epigenetic implications in terms of nucleosome positioning, as well as three-dimensional chromatin organization and functions. Captivating findings have indeed confirmed that G4-forming sequences are located within non-nucleosomal DNA regions in organisms as diverse as yeasts, nematodes, mice, and humans [62,172,173,174,175]. The hypothesis that G4 structures may function as nucleosome exclusion signals is further supported by the observation of inverse phasing patterns between G4 motifs and nucleosomes around a subset of human DNA replication origins [176]. More specifically, the nucleosome-depleted regions containing G4-forming sequences quite frequently coincide with the boundaries of topologically associated domains [150]. These consist of nucleoprotein complexes highly enriched in architectural proteins, such as CTCF and cohesin, involved in three-dimensional partitioning of the eukaryotic genome and correlated with gene regulation [177,178,179,180,181]. Otherwise stated, there is consistent evidence suggesting that G4s may mediate the preferential establishment of long-distance contacts between different regions of interphase chromosomes (Figure 3C). While doing so, G4s could also affect gene expression by facilitating interaction between promoters and their cis-regulatory sequences via looping [150,182]. In this regard, it is worth noting that the so-called “half G4s” are significantly enriched exactly at the abovementioned genomic regions [182]. Individual half G4 sequences are unable to fold into autonomous G4 structures, being composed by only two shortly interspaced runs of at least three consecutive guanines. Nonetheless, two distinct half G4 may join into an intermolecular G4 structure, thereby bringing together two distant genomic regions [182].

### 3.3. Epigenetic Roles of G4 Structures Formed in Coding and Noncoding RNA Transcripts

Although, to date, structural and functional studies have been mainly focused on DNA G4s, in the last decade, there has been growing attention on RNA G4s in light of the wide variety of functions attained by these structures in multiple physiopathological processes. A fundamental concept that has emerged from several studies is that RNA and DNA G4 structures, despite numerous similarities, are not merely counterparts of each other. In fact, due to the presence of a C2′-hydroxyl group in the ribose sugar and uracil in the spacer loops, RNA G4s are often more compact, less hydrated, and more thermodynamically stable structures than their DNA analogs [183,184,185,186]. On the other hand, RNA G4s almost exclusively adopt a monomorphic parallel topology due to the steric constraints imposed by sugar puckering and C2′-hydroxyl groups, which lock the riboguanosines in an *anti* conformation [187]. Within eukaryotic cells, RNA G4s are widely distributed in both nuclear and cytoplasmic compartments, implying that they have a greater assortment of protein-binding partners compared to DNA G4s and that they can potentially fold into hybrid intermolecular RNA:DNA structures in the nucleoplasm [30,188].

**Figure 4:**
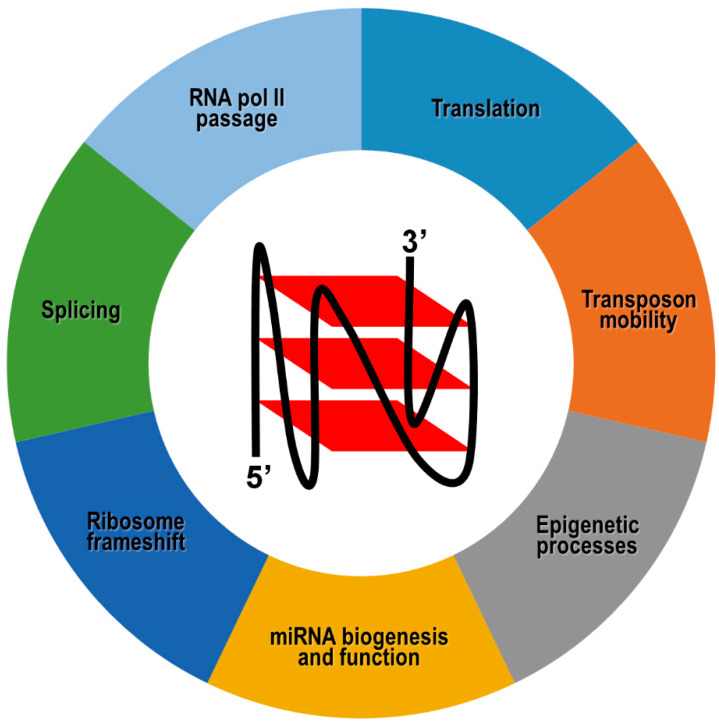
Molecular processes modulated by G4 structures formed in coding and noncoding RNA transcripts. A schematic drawing indicating the parallel topology adopted by RNA G4s is shown.

In general, switchable RNA G4s potentially provide an important contribution to epigenetic regulation of gene expression, and their involvement in a given biological function is strictly context-dependent and antagonized by specialized helicase activities (Figure 4). For example, negative cotranscriptional regulation of reporter gene expression by an impediment to RNA polymerase passage has been reported following the formation of hybrid RNA:DNA G4s between the nascent RNA and the template, but not the coding, DNA strand [189]. As already mentioned, this mechanism could be further refined by dynamic G4 unfolding and refolding governed by helicases. However, in silico predictions have revealed that this cotranscriptional mechanism could be underrepresented in warm-blooded animals, whose genes exhibit a strong bias toward G4 motifs on the coding DNA strand, and therefore, on the corresponding mRNA [190].

In this case, the differential location of G4s in exons, introns, 5′- and 3′-UTRs may exert totally distinct outcomes on gene expression. More specifically, G4s located in 5′-UTR of coding transcripts normally inhibit translation initiation, probably through sterically hindering the recruitment and/or scanning of the 40S ribosome subunit on the mRNA [191,192,193,194,195]. Based on these findings, and considering that G4s have been identified in the 5′-UTR of numerous proto-oncogene transcripts, it has been proposed that such RNA motifs could be suitable targets for small molecules, with the view to interfering with gene expression at the translation level [196]. Indeed, small molecules could, in principle, affect G4 stability and/or compete with G4-binding proteins normally involved in translation stimulation. However, there are no approved anticancer drugs targeting RNA or DNA G-quadruplexes, probably due to nonspecific binding events of small molecules to nucleic acid structures other than G4s.

In a few alternative cases, G4 structures present in 5′-UTR were proposed to be necessary for atypical forms of translation, although the molecular mechanisms underlying these events are not completely understood [197,198,199,200,201,202,203]. Formation of G4 structures at the exon-intron boundaries or near to polyadenylation signals of pre-mRNAs may affect either positively or negatively the binding of regulatory proteins, ultimately leading to the expression of alternative protein isoforms [204,205,206,207]. Similarly, G4 structures within mRNAs may act as translational recoding signals, forcing the ribosome to shift one ribonucleotide backward with respect to the former open reading frame, thereby decoding a distinct protein [208,209].

Another exquisitely epigenetic function accomplished by RNA G4s pertains the transport of mRNAs to subcellular compartments, which allows localized protein synthesis. In particular, G4 structures located at the 3′-UTR represent a very common and effective dendritic localization signal of several neuronal mRNAs that travel in a centrifugal direction along axons. G4 structure stability being differentially influenced by monovalent cations, it has been proposed that sudden variation of intracellular K^+^ and Na^+^ concentrations occurring during neuronal activity could govern G4 folding/unfolding, thereby controlling protein-dependent transport of these dendritic mRNAs and translation at their subcellular destination [210,211,212,213,214].

G4 structures have also been mapped in short and long noncoding RNAs, although their biological implication is still unclear in most circumstances [215,216]. Bioinformatics analysis revealed that about 16% of human microRNA precursors present G4s either in the passenger or guide strand [217]. Overall, these G4s could exist in equilibrium with stem-loop secondary structures typically formed by pre-miRNAs, thereby playing a role in microRNA biogenesis by modulation of Dicer cleavage [217,218,219]. In the case of pre-miR-26a-1, G4 unwinding achieved by the RNA helicase DHX36 favors the physiological accumulation of mature miR-26a, and impairing DHX36 activity leads to low miR-26a abundance and obesity [220]. It has also been reported that G4 structures are retained in a number of biologically relevant human microRNAs in their mature form, suggesting that G4 formation could impact microRNA-directed post-transcriptional regulation of gene expression by affecting microRNA-target mRNA interactions [221].

Recent works have also shown that RNA G4s are involved in the regulation of transposable genetic elements mobility. Indeed, human retrotransposon remnants belonging to the LINE-1 family harbor distinct types of G4-forming sequences in their 3′UTR and there is a positive correlation between G4 structure stability and LINE-1 mobilization activity [222]. On the other hand, separate findings have shown that piR-48164, belonging to the PIWI-interacting family of human small noncoding RNAs, similarly bears a stable G4 structure [223]. Once formed, such an RNA G4 is detrimental to PIWI binding and prevents the piR-48164-directed transposon control at the transcriptional and post-transcriptional level [223]. 

In addition to these mechanisms occurring on RNA, multiple studies have suggested that G4 formation in long noncoding RNAs affect epigenetic processes on DNA, as demonstrated for G4s at telomeric repeat-containing RNAs (TERRAs) [224,225]. Indeed, it was noted that G4 structures formed by TERRAs specifically recruit the histone H3K4-specific demethylase LSD1, which is associated with telomeric heterochromatinization [151].

## 4. Conclusions and Perspectives

G4 structures have been extensively studied both in silico and in vitro, and in recent years have witnessed a considerable increase in experimental data, substantiating the versatile epigenetic roles of these unconventional structural motifs within cells. In a nutshell, G4s are widespread throughout the genome and transcriptome of various organisms, where they overall influence DNA and histone modifications, nucleosome positioning, and three-dimensional organization of chromatin, as well as post-transcriptional modulation of gene expression. Therefore, it could be argued that context-specific formation of G4s in the genome might direct when and where epigenetic modifications are imposed. On the other hand, the concerted balance between G4 formation/disruption controlled by helicases during DNA replication aids in preserving epigenetic memory. Unfortunately, whether and how G4-mediated epigenetic marks imposed during post-replication are linked to the next round of DNA duplication remains to be confirmed. Similarly, another challenging issue so far unexplored pertains to the potential relationship between G4 and transgenerational inheritance of epigenetic information via the germline of organisms with sexual reproduction.

As discussed, G4 structures are recognized by a plethora of *trans*-acting factors, which can modulate their stability or serve as a scaffold for the recruitment of further epigenetic effectors. These findings, coupled with the insights linking G4 deregulation to numerous human diseases, suggest promising therapeutic interventions. In this respect, several classes of small molecule ligands have been described to induce G4 conformations and modulate G4–protein interactions. However, further investigations are required to overcome nonspecific binding events of G4 ligands to nucleic acid structures other than G4s. A better understanding of the exact targets of these ligands will potentially improve bona fide patient-specific clinical applications. In parallel, the multitude of G4-interacting proteins could be used to design novel drug molecules. Most probably, the combined use of ligands directed against G4 structures and G4-interacting proteins could be even more effective and specific for therapeutic purposes. However, the epigenetic effects of all these known and potential molecules remain to be confirmed in physiologically relevant settings. From this standpoint, an advancement of in vivo studies implementing suitable animal models is absolutely required for a comprehensive understanding of biological roles played by G4s in both normal and pathological contexts. 

## Abbreviations

5meC	C5-methyl-deoxycitosine
6meA	N6-methyl-deoxyadenosine
6meG	O6-methyl-deoxyguanine
8oxoG	8-oxo-7,8-dihydro-guanine
APE1	apurinic-apyrimidinic endonuclease 1
ASXL1	Additional Sex combs-Like 1
ATRX	ATP- dependent X-linked helicase
BRD3	Bromodomain Containing 3
CTCF	CCCTC-binding Factor
DAXX	Death Domain Associated Protein
DHX36	DEAH-Box Helicase 36
DNMT	DNA methyltransferase
FACT	Facilitates Chromatin Transcription
FANCJ	Fanconi Anemia group J
FUS	Fused in Sarcoma protein
G4	G-quadruplex
HIF1-α	Hypoxia-inducible factor 1-α
HMGB1	Chromosomal High Mobility Group Box 1
hTERT	human Telomerase Reverse Transcriptase
KMT5C	Histone-lysine N-Methyltransferase KMT5C
LINE-1	Long Interspersed Nuclear Element-1
LSD1	Lysine Specific Demethylase 1
lysC	lysozyme C
NuRD	Nucleosome Remodeling and Deacetylase complex
OGG1	8-oxoguanine DNA glycosylase
PADi4	Peptidylarginine Deiminase 4
PARP1	Poly ADP-Ribose Polymerase 1
PHF8	Histone Lysine Demethylase 8
PRC2	Polycomb Repressive Complex 2
REST	RE1-Silencing Transcription Factor
SWI/SNF	SWItch/Sucrose Non-Fermentable
TERRA	Telomeric Repeat-containing RNA
TLS	Translocated in Liposarcoma protein
UTR	Untranslated Region
VEGF	Vascular Endothelial Growth Factor
